# Wilson Disease: Update on Pathophysiology and Treatment

**DOI:** 10.3389/fcell.2022.871877

**Published:** 2022-05-02

**Authors:** Som Dev, Robert L. Kruse, James P. Hamilton, Svetlana Lutsenko

**Affiliations:** ^1^ Department of Physiology, Johns Hopkins Medical Institutes, Baltimore, MD, United States; ^2^ Department of Pathology, Brigham and Women’s Hospital, Boston, MA, United States; ^3^ Department of Medicine, Johns Hopkins Medical Institutes, Baltimore, MD, United States

**Keywords:** copper, liver, Wilson disease, ATP7B, nuclear receptor

## Abstract

Wilson disease (WD) is a potentially fatal genetic disorder with a broad spectrum of phenotypic presentations. Inactivation of the copper (Cu) transporter ATP7B and Cu overload in tissues, especially in the liver, are established causes of WD. However, neither specific ATP7B mutations nor hepatic Cu levels, alone, explain the diverse clinical presentations of WD. Recently, the new molecular details of WD progression and metabolic signatures of WD phenotypes began to emerge. Studies in WD patients and animal models revealed the contributions of non-parenchymal liver cells and extrahepatic tissues to the liver phenotype, and pointed to dysregulation of nuclear receptors (NR), epigenetic modifications, and mitochondria dysfunction as important hallmarks of WD pathogenesis. This review summarizes recent advances in the characterization of WD pathophysiology and discusses emerging targets for improving WD diagnosis and treatment.

## Introduction

Wilson disease (WD) is an autosomal-recessive disorder of copper (Cu) metabolism caused by inborn mutations in the Cu(I) transporting ATPase beta polypeptide (ATP7B). Mutations in ATP7B disrupt Cu homeostasis, causing Cu accumulation in the liver and other tissues (Członkowska et al., 2018). Hepatic manifestations of WD range from asymptomatic elevation of hepatic transaminases to fibrosis, cirrhosis, and acute liver failure (Członkowska et al., 2018). Although WD is a monogenic disorder, the time of disease onset and specific presentations vary significantly, which points to existence of modifying factors (Schiefermeier et al., 2000; Ferenci 2014; Ferenci et al., 2019). Variable clinical presentations make the diagnosis, treatment and the mechanistic understanding of WD challenging (Roberts 2018; Stättermayer et al., 2019).

WD has an asymptomatic stage, when hepatic Cu is already elevated, but liver morphology and function are not yet significantly compromised owning to an upregulation of Cu-sequestering metallothioneins and an increased glutathione synthesis. With time, accumulating metabolic and transcriptional changes, oxidation and other posttranslational modifications overwhelm these protective mechanisms, triggering histologic abnormalities, increased autophagy, and diminishing liver function, without further increases in hepatic copper. Down-regulation of hepatic CTR1 and hepatocyte death cause Cu to be diverted from the liver into the circulation, accelerating Cu accumulation in other tissues (Gray et al., 2012) and triggering neurological and psychiatric disturbances (Członkowska et al., 2018). While this sequence of events in WD has long been established, the molecular basis of underlying pathologic changes at each step of the disease are only now emerging; this progress has been accelerated by the availability of several animal models of WD (Reed et al., 2018).

Animal studies demonstrate that multiple cellular compartments (nuclei, mitochondria, lysosomes, autophagosomes) participate in hepatocytes response to Cu overload, and multiple pathways are involved. Inhibition of nuclear receptors (Wooton-Kee et al., 2015; Hamilton et al., 2016; Wooton-Kee et al., 2020), epigenetic modifications (Mordaunt et al., 2018; Mordaunt et al., 2019; Sarode et al., 2021) and mitochondria dysfunction (Roberts et al., 2008; Zischka et al., 2011) have been identified as important hallmarks of the disease. Increased autophagy was observed in Atp7b^−/−^ deficient cells and in the livers of WD patients (Polishchuk et al., 2019). The metabolomic analysis of WD patients’ serum suggested existence of distinct metabolic profiles differentiating WD from other liver disorders as well as WD with different phenotypic manifestations (Sarode et al., 2019; Azbukina et al., 2020). The contribution of miRNA and long non-coding RNA to pathogenesis of WD appears likely (Zhang et al., 2021), but remains understudied. Significant gender-related differences in Cu levels, metabolic, and fibroinflammatory changes are observed in human WD and WD mouse models emphasizing the importance of including both sexes in testing new therapeutic approaches (Litwin et al., 2012; Li et al., 2018; Gottlieb et al., 2022). In this review, we summarize recent developments in understanding of WD pathogenesis and progress towards the next generation of diagnostics and therapeutics.

### Copper Homeostasis in the Liver

**FIGURE 1:**
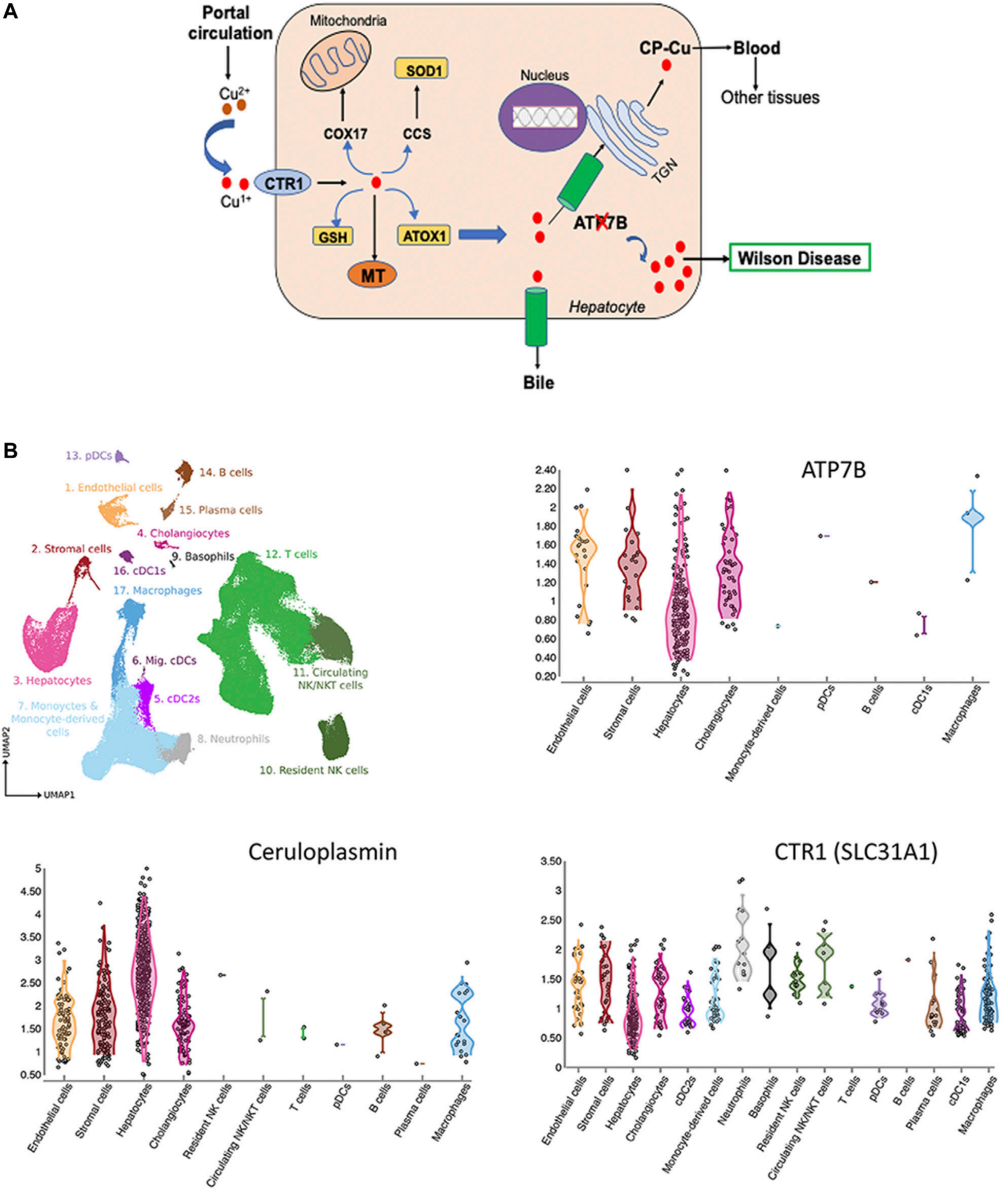
Cu homeostasis in liver **(A)** and Cu-handling proteins in liver cells **(B). (A)** Cu enters liver via portal circulation and transported into liver cells primarily by the high affinity uptake protein, CTR1. Cytosolic Cu chaperones shuttle Cu to specific intracellular targets; CCS transports Cu to SOD1, ATOX1 - to the Cu-transporting ATPase ATP7B. ATP7B transports Cu into the trans-Golgi network (TGN) for incorporation into ceruloplasmin (CP) and to the apical membrane for excretion. Inactivation of ATP7B causes Cu overload, which manifests clinically as WD (ATP7B-ATPase Cu(I) transporting beta polypeptide; CTR1-high affinity Cu uptake protein 1; MT-Metallothionein; GSH-Glutathione, ATOX1-antioxidant protein 1; SOD1-Superoxide dismutase; CCS-Cu Chaperone for SOD, COX17-Cytochrome C oxidase) (Lutsenko 2016; Członkowska et al., 2018). **(B)** Expression of ATP7B, CP and CTR in liver cells. The figure is generated using Liver Cell Atlas (https://www.livercellatlas.org/), which aggregates single cells sequencing data for human and animal livers.

Cu is essential micronutrient for human growth and development, and liver is the major Cu homeostatic organ in humans and animals (Roberts and Sarkar 2008). Cu homeostasis in the liver is maintained by the network of proteins, which include transmembrane Cu transporters (CTR1 and ATP7B), cytosolic Cu carriers (chaperones), Cu storage proteins (metallothioneins) and Cu-requiring enzymes (Figure 1A). Several liver enzymes use Cu for their activity: the ferroxidase ceruloplasmin (CP), an abundant Cu-binding protein secreted by hepatocytes into the blood (Linz and Lutsenko 2007), cytochrome c oxidase (mitochondrial respiration), superoxide dismutase 1 (free radical defense), factor VIII (blood clotting) and other less abundant proteins.

ATP7B is central for liver Cu homeostasis. It delivers Cu from the cytosol to CP in the *trans-*Golgi network; and when Cu is elevated, ATP7B traffics towards the apical membrane to facilitate Cu export into the bile. Inactivation of ATP7B disrupts these processes, causing Cu accumulation in the liver and secretion of apo-CP, which is unstable and inactive (Merle et al., 2010; Merle et al., 2009). It is firmly established that ATP7B is expressed in hepatocytes; however, single cell sequencing studies revealed additional information about the Cu-handling machinery of various cells types in the liver. Based on CTR1 expression, hepatocytes, macrophages, cholangiocytes, and stromal cells are the main importers and users of Cu (Figure 1B). In addition to hepatocytes, ATP7B is present in cholangiocytes, endothelial, and stromal cells and its expression parallels expression of CP (Figure 1B). The ATP7B homologue, ATP7A, which was thought not to be expressed in the liver, has now been found in most cells, including hepatocytes (https://www.livercellatlas.org/). Although ATP7A does not compensate for the loss of ATP7B function, ATP7A can be induced in the liver in response to signaling from other tissues and facilitate Cu export (Kim et al., 2010). The signaling molecule that upregulates hepatic ATP7A remains elusive, but identification of this molecule may be the first step towards de-coppering liver in WD using endogenous means.

### Wilson Disease Liver Phenotype is Determined by Copper Misbalance in Different Cell Types in the Liver

Comparison of mice with a global inactivation of Atp7b (as in human WD) and hepatocyte-specific inactivation (Atp7b△Hep) highlighted the contribution of various liver cell types to WD pathogenesis (Muchenditsi et al., 2017; Muchenditsi et al., 2021). The Atp7b△Hep mice accumulate Cu in the liver, produce apo-CP, but show no ballooning (apoptotic) hepatocytes nor inflammation, which are commonly seen in global Atp7b knockouts (Atp7b^−/−^ mice) and in human WD. The only obvious pathology in Atp7b△Hep animals is liver steatosis (Muchenditsi et al., 2017). This finding harmonizes with clinical data showing steatosis to be an early disease manifestation and further suggests that the development of inflammatory responses in WD may depend on the Cu status of non-parenchymal liver cells. Indeed, non-parenchymal liver cells in Atp7b△Hep mice have normal Cu levels in contrast to elevated Cu in non-parenchymal cells in Atp7b^−/−^ mice (Muchenditsi et al., 2017). Proteomics studies show that when Atp7b inactivation is limited to hepatocytes, the liver upregulates proteins involved in redox balance, mitochondria function, amino-acid and lipid metabolism; all of which compensates for functional deficiencies caused by Cu overload (Muchenditsi et al., 2021). This compensatory capacity is lost in Atp7b^−/−^ mice leading to significant metabolic disturbances and activation of energy sensor, AMP kinase (Wooton-Kee et al., 2020). In addition, inactivation of ATP7B in the intestine dysregulates the dietary fat processing and chylomicron assembly and may exacerbate metabolic disturbances in the liver (Pierson et al., 2018). Further studies are needed to better understand the role of non-parenchymal liver cells and extrahepatic tissues in human WD.

### Epigenetics and Modifying Factors in Wilson Disease

Lack of strong genotype-to-phenotype correlations in WD reflects the influence of environmental and epigenetic factors. Several genetic modifiers of WD are proposed based on studies of gene allele frequencies in WD patients along with dietary factors that may influence the disease progression (reviewed in (Kieffer and Medici 2017; Medici and Weiss 2017)). In the rat model of WD, high calorie diet accelerated liver failure (Einer et al., 2019), whereas in the mouse model of WD on a similar diet, inflammatory response was diminished in favor of steatosis (Wooton-Kee et al., 2020; Gottlieb et al., 2022). This finding in mice could be linked to activation of mTORC1 and inhibition of autophagy, and further studies can test this hypothesis. Studies in animals also suggest that natural differences in levels of Cu-chelating metallothioneins MT1/2 may result in different capacities to buffer accumulated Cu and thus modulate liver response to excess Cu (Muchenditsi et al., 2021). In the future, it would be interesting to determine whether the levels of metallothioneins in humans can be used as predictors of the timing of disease onset and progression. ATOX1, COMMD1, and XIAP were shown not to contribute significantly to the WD phenotype (Kieffer and Medici 2017), whereas polymorphisms in PNPLA3, a lipase involved in hepatocyte triglyceride metabolism, were associated with increased hepatic steatosis in WD (Stättermayer et al., 2015). Hydroxysteroid 17-β dehydrogenase polymorphisms in WD appear to have protection against acute liver failure (Pop et al., 2021).

Epigenetic modifications influence gene expression without altering DNA sequences. The best understood epigenetic mechanisms are DNA methylation (Jones 2012) and acetylation (Ganesan et al., 2009). Methylation involves the addition of methyl groups to cytosine bases, typically at CpG sites (Bender 2004) and is mediated by DNA methyltransferases (Okano et al., 1999). Recent studies provide strong evidence for contribution of epigenetics to WD pathogenesis. Aberrant DNA methylation and abnormal 1-carbon metabolism is reported in WD patients and animal models of WD (Medici and LaSalle 2019; Mordaunt et al., 2019). DNA methylation is highly dependent on the availability of the universal methyl donor S-adenosyl-methionine (SAM). Cu inhibits the activity of S-adenosyl-L-homocysteine hydrolase (Li et al., 2007), a key enzyme that regulates the amount of SAM available for methylation, which may in part explain hypomethylation, although the overall mechanism is likely to be more complex. SAM levels are also affected by dietary uptake of folate, vitamin B12, methionine, betaine and choline, as well as genetic variations in enzyme mediating one-carbon metabolism (Niculescu and Zeisel 2002). Dietary supplementation of methyl donor such as betaine (Medici et al., 2013) or choline (Medici et al., 2016) demonstrated that changes in a global DNA methylation status in Atp7b-deficient liver can be reversed.

Studies of DNA methylation in human WD revealed differentially methylated region (DMRs) in liver samples (Mordaunt et al., 2019). The WD-specific DMRs were associated with genes enriched in lipid, folate metabolism and inflammatory response. Genes associated with response to oxidative stress (such as Hif1, Gstp1, and thioredoxin) were differentially methylated in human WD liver (Mordaunt et al., 2018). DNA methylation signatures could be one of the potential biomarkers and/or therapeutic targets.

Histone acetylation (HA) is a dynamic epigenetic modification that regulates transcription, and it is also impaired in murine WD (Sarode et al., 2021). HA is controlled by histone acetyltransferases and deacetylases (Ganesan et al., 2009). A significant decrease in histone deacetylases 4 and 5 (HDAC4/5) is observed in the tx-j mouse model of WD (an inbred mouse strain with a missense mutation in Atp7b) (Sarode et al., 2021). H3ac, H3K9ac, and H3K27ac levels are increased in livers of tx-j mice and supplementing these animals with diets enriched with methyl donors or Cu chelation restored levels of HDAC4/5 (Sarode et al., 2021). These findings may help to understand epigenetic modifications (acetylation and methylation) observed in WD and other liver disorders with similar presentation (Dev and Hamilton 2021).

### Nuclear Receptor Dysfunction in Wilson Disease

Nuclear receptors (NR) are ligand dependent transcription factors that regulate gene expression of multiple signaling pathways. Regardless of clinical presentation, hepatic Cu is a hallmark of WD and it causes NR inhibition (Wooton-Kee et al., 2015). Defects in NR signaling alter lipid metabolism in WD patients and Atp7b^−/−^ mice (Wilmarth et al., 2012; Wooton-Kee et al., 2015; Hamilton et al., 2016). Reduced activity of LXR, FXR, RXRα, HNF4α, LRH-1 and PPARα link nuclear receptor dysfunction to WD (Nagasaka et al., 2012; Wooton-Kee et al., 2015; Wooton-Kee et al., 2020). Alterations in NR activity differ at different stages of WD. LXR/RXR was identified as one of the major targets of elevated Cu, especially early in the disease (Hamilton et al., 2016). In the mouse model of WD, LXR is inhibited at 6 weeks after birth, which is an asymptomatic stage of the disease (Hamilton et al., 2016), and other NR receptors become dysregulated as the disease progress (Nagasaka et al., 2012; Wilmarth et al., 2012; Wooton-Kee et al., 2015). Cu does not alter LXR protein levels or blocks its ability to bind substrates. Accordingly, treatment with a LXR agonist (in Atp7b^−/−^ WD mice) prevents injury even in the presence of high Cu (Hamilton et al., 2016). Further studies are needed to determine the contribution of LXR and other NR dysfunction to inflammation and fibrosis, especially in humans. The activity of nuclear receptors in Atp7b^−/−^ liver may reflect a complex interplay of metabolites generated by the Cu-altered enzymes as well as transcriptional activities of NRs *per se*. For example, copper induced oxidative stress and its downstream effect on generation of LXR ligands and antagonists is a possible explanation for downregulation of LXR signaling. Studies with LXR agonists in WD models will better define LXR dependent pathways in WD and may lead to new therapies targeting these pathways (see below).

### Available and Emerging Treatments

In WD, phenotypic heterogeneity and lack of unique manifestations can present diagnostic and treatment challenges. Current treatments include Cu chelation, zinc salts, and liver transplantation. Cu chelation is the standard-of care therapy for WD and provides a significant benefit for most patients, especially if initiated early (Czlonkowska et al., 2014). D-penicillamine and Trientine are both approved for use in WD by most regulatory drug agencies, while Tetrathiomolybdate is approved for use in Europe and in a Phase III trial in the United States. Zinc acetate and other zinc salts regulate body Cu balance by presumably decreasing Cu absorption (Czlonkowska et al., 2014). Zinc salts are typically used in pre-symptomatic patients, and as a maintenance drug after chelation. Combination of chelation and zinc salts is common in clinical practice, but not well studied. Despite proven benefits, current therapies have limitations, including side effects, poor compliance, high cost (up to $300,000 per yer), and potential for neurological decompensation (Merle et al., 2007; Schilsky et al., 2015). A significant percentage of WD patients with primarily neurologic manifestations do not respond well to treatment and these patients are at high risk for deterioration (Mohr and Weiss 2019). Monitoring Cu levels on treatment requires calculation of non-ceruloplasmin bound copper content in serum, which can be challenging. Novel methods of measuring free copper using anion-exchange chromatography coupled to triple quadrupole inductively coupled plasma mass spectroscopy are in development and look very promising (Solovyev et al., 2020).

In WD patients with acute liver failure, liver transplantation remains the only treatment option. Recent studies in the rat model of WD, found that methanobactin, a peptide produced by proteobacterium Methylosinus *trichosporium*, can be successfully used to remove excess Cu from mitochondria, decrease liver histopathology, and prevent liver failure (Lichtmannegger et al., 2016). Mitochondria disfunction is one of the important hallmarks of WD in human and animals (Sternlieb 1968; Roberts et al., 2008; Zischka et al., 2011), and therefore these pre-clinical results are significant. At the same time, developing precise dosing and a treatment regimen may be challenging, because methanobactin has high affinity for Cu and un-intended over-depletion of Cu in the mitochondria may disrupt the respiratory chain and be as harmful as Cu overload. Short-term treatment with subsequent zinc maintenance could be considered. Methanobactin has not yet been studied in humans.

Since dysregulation of nuclear receptors, especially LXR and FXR, contribute significantly to WD phenotype (see above), targeting these receptors, could be an attractive alternative option for patients who do not respond or poorly tolerate Cu chelation. Strong evidence exists that LXR is inhibited in Atp7b^−/−^ liver, and that treatment with the LXR agonist T0901317 significantly delays the pathology onset and improves liver function in mice (Hamilton et al., 2016). However, in pre-clinical studies unrelated to WD, T0901317 was shown to induce hepatic steatosis and hypertriglyceridemia making it unsuitable candidate for treating WD. Further studies are needed to clarify the usefulness of this and/or other LXR agonists for treating human WD.

An exciting potential treatment for WD is delivering a functional ATP7B gene (cDNA) into WD patients. While the premise of gene therapy for WD is straightforward, there are two major hurdles for application to WD. The first consideration is that WD is a systemic disease with ATP7B expression in multiple cell types. While gene therapy vectors can be delivered systemically and enter multiple cell types, the majority of viral vectors and resultant expression resides in the liver (Wang et al., 2019). Thus, current WD gene therapy should primarily be considered a liver-specific correction of the disorder. While this liver-specific expression is a limitation, case reports of liver transplantation reversing neurologic WD suggest that liver-specific expression could be sufficient for some WD patients (Catana and Medici 2012; Poujois et al., 2020). The second consideration is that the cDNA for ATP7B is large (approximately 4.4 kilobases, even without the promoter and polyA sequences), and when the full-length ATP7B packaged into the most common gene therapy vector, adeno-associated virus (AAV), the production yields of virus are low (Murillo et al., 2016). To address this problem, miniature ATP7B (miniATP7B) was developed by deleting the first four metal binding domains from ATP7B (Leng et al., 2019; Murillo et al., 2019). The miniATP7B was shown *in vitro* to have ATP7B activity (Leng et al., 2019; Murillo et al., 2019); however, precise intracellular regulation is lost when the first 4 metal-binding domains are deleted (Jayakanthan et al., 2017).

The most successful WD gene therapy studies have used AAV vectors in WD mouse models. Using liver-specific promoters, initial studies found that administration of AAV before the onset of liver pathology was effective for partial (Greig et al., 2019) or full disease (Murillo et al., 2016) reversal. Higher doses of AAV are required if liver injury is already present, which would be the case in most WD patients (Murillo et al., 2019), and presence of fibrosis is especially challenging. Concerning the delivery efficiency required to reverse WD, one study found that approximately 20% hepatocyte expression of ATP7B could reverse all markers of WD. It remains unclear whether it is sufficient to correct Atp7b in hepatocytes alone or is correction in non-parenchymal liver cells also needed, and the applicability of this approach in humans.

Two Phase I/II AAV gene therapy trials are currently underway for WD (NCT04537377, Vivet Therapeutics; NCT04884815, Ultragenyx). Translating efficacy of gene therapy in mice to human patients has proved challenging, thus success is not ensured. For example, gene therapy for hemophilia results in a 100-fold loss in gene expression per AAV dose (Nathwani et al., 2006; Nathwani et al., 2011). Thus, significantly higher doses may be required in WD patient trials versus WD mouse models. Another concern is that the liver injury and hepatocyte turnover in WD could lead to dilution of the episomal AAV vector as cells divide, raising uncertainty of how long the AAV therapeutic effect may last. These questions will hopefully be answered once the first data is released from these clinical trials.

An alternative strategy to AAV is ATP7B-carying lentiviruses administered into WD mice during gestation. This method of gene treatment improved liver histology and hepatic Cu content was reduced, but did not uniformly normalize Cu levels and variable ATP7B expression was observed (Roybal et al., 2012). CRISPR-mediated correction could be considered for WD, but the hundreds of different mutations in ATP7B and their often compound heterozygous nature complicate site-specific correction with gene editing (Pöhler et al., 2020).

**FIGURE 2:**
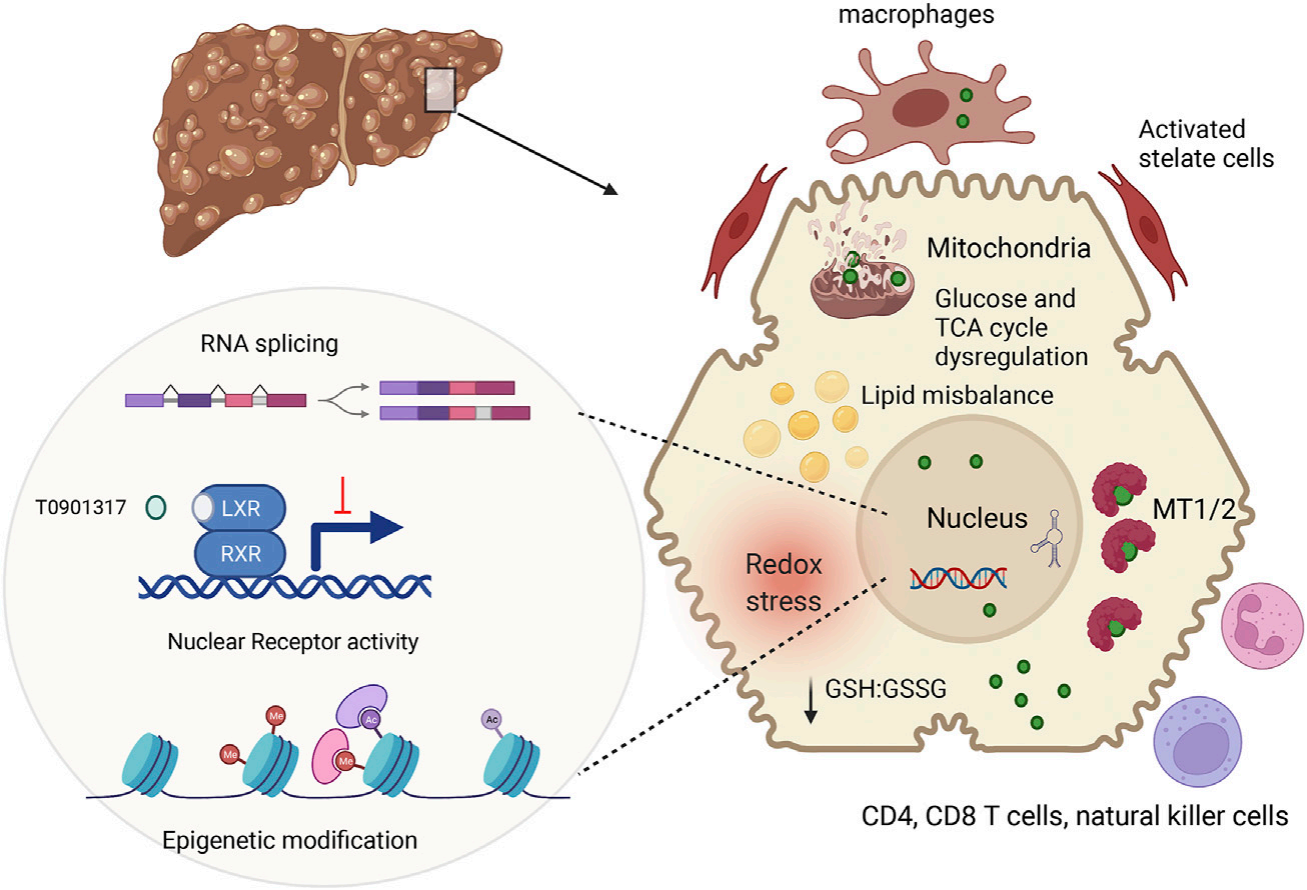
Summary of main pathologic changes in WD liver. ATP7B mutations result in hepatic accumulation of copper (green circle). In the cytosol, Cu is sequestered by metallothioneins (MT1/2), whereas excess Cu causes glutathione oxidation (lower GSH:GSSG ration), contributing to redox stress. Cu elevation in nuclei, alters RNA processing, including splicing (Burkhead et al., 2011) inhibits NR function and induces epigenetic changes. Downstream effects include dysregulation of metabolic profiles in hepatocytes. Hepatocyte injury and possibly Cu accumulation in non-parenchymal cells stimulates immune cells and stellate cells, resulting in inflammation and fibrosis. The figure was generated using BioRender.

In conclusion, recent studies in WD patients, murine WD models, and cell lines with inactivated ATP7B have significantly expended and deepened our understanding of WD pathophysiology (Figure 2). These new findings suggest that specific biomarkers and improved treatments can eventually be developed for WD with different disease manifestations.
